# The history of haploidentical stem cell transplantation: a trip from the bench to the bedside

**DOI:** 10.1080/16078454.2024.2346401

**Published:** 2024-04-30

**Authors:** Mariana G. Meade, Javier Bolaños-Meade

**Affiliations:** a Johns Hopkins University; b Johns Hopkins University School of Medicine, Clinical Director, BMT Program, The Sidney Kimmel Comprehensive Cancer Center at Johns Hopkins, Baltimore, Maryland, USA

**Keywords:** Haploidentical, HLA-matched, bone marrow transplantation, acute graft versus host disease, chronic graft versus host disease, graft failure, alternative donors, post-transplantation cyclophosphamide

## Abstract

Allogeneic bone marrow transplantation is a curative intervention for both neoplastic and non-malignant conditions. However, not all patients have an HLA-matched donor. Therefore, the development of an approach that expand the donor pool was of paramount relevance. The development of post-transplantation cyclophosphamide as graft versus host disease prophylaxis allows the safe use of haploidentical donors, solving the donor availability problem to the vast majority of patients in need. The present paper reviews the history of the development of haploidentical transplantation at Johns Hopkins University, from the bench to the bedside.

## Introduction

**Figure 1.**
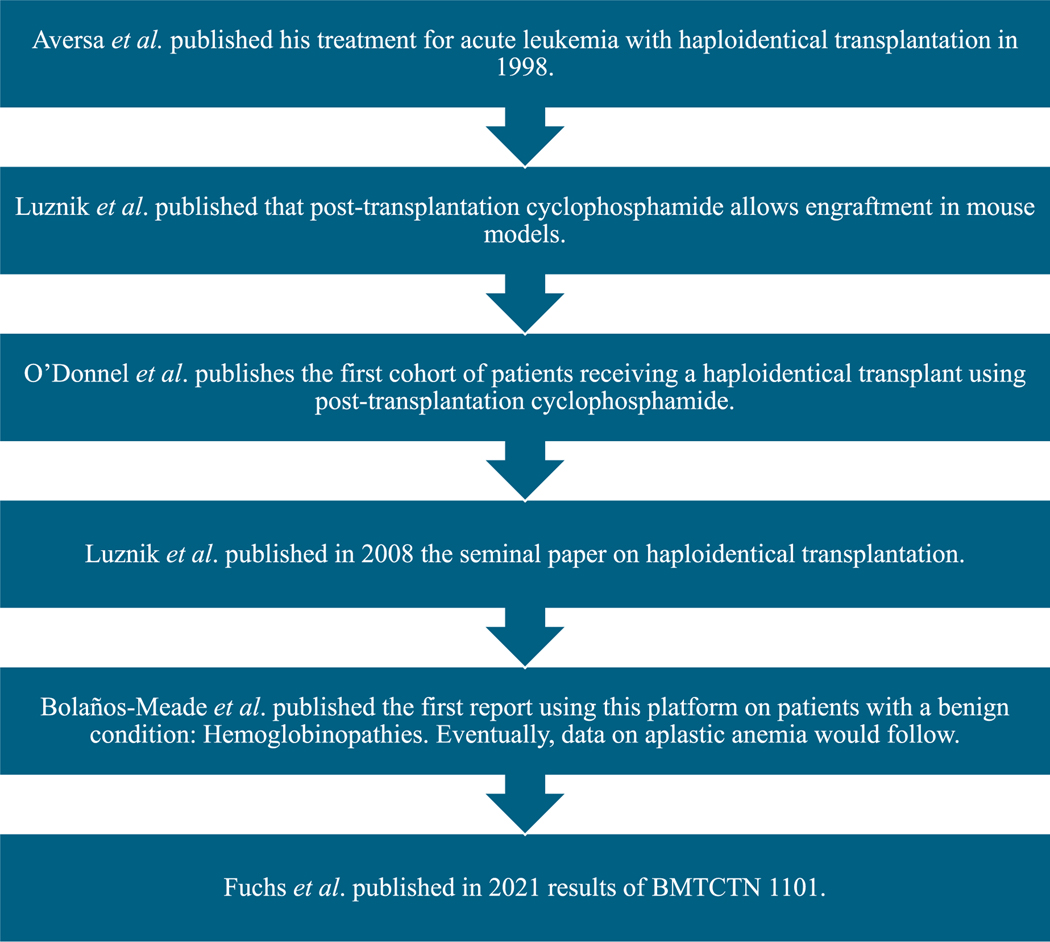
Historical development of haploidentical transplantation utilizing posttransplantation cyclophosphamide.

Allogeneic bone marrow transplants (BMT) can serve as a cure for a large number of diseases in scenarios where chemotherapy is not enough. However, it presents challenges due to the small size of the donor pool as well as the risk for toxicities such as graft-versus-host disease (GVHD). Ideally, one would like to find a human leukocyte antigen-matched (HLA-matched) donor, as it has historically lowered the risk of GVHD or rejection and produced better long-term outcomes [1]. However, the use of reduced-intensity conditioning (RIC) has allowed for haploidentical stem cell transplantation to have a lower prevalence of toxicities as well as higher rates of survival. In this manuscript, we do not intend to review all studies ever published with haploidentical transplantation exhaustively, but to give the reader an idea of how the concept evolved from the laboratory, and was translated to the clinic, how it fares when compared to other approaches, as well as specific deviations from the original schema decreasing the immunosuppression or its use in nonmalignant hematology. This is a review of the journey over the last 25 years of haploidentical transplantation at Johns Hopkins University (see Figure 1).

### Pre-clinical experience

HLA-matched siblings historically presented as the best option in finding a donor for BMT as historically, they result in the better outcomes regarding GVHD, overall survival (OS), and progression-free survival (PFS). However, this option is not available to all patients, presenting them with the choice of an unrelated donor, umbilical cord blood, or partially HLA-matched or HLA-haploidentical donors. In the 1970s, Korngold *et al.* found that T-cell depletion (TCD) prevented GVHD after an allogeneic BMT in mice; this was translated to the clinic by Aversa *et al.* who published a series of studies utilizing TCD in patients undergoing haploidentical BMT (Haplo-BMT) [2–5]. TCD reduced instances of GVHD at the cost of higher rates of graft failure (GF), disease relapse, and non-relapse mortality (NRM). In an attempt to overcome these problems, new techniques were needed. In the laboratory, Luznik *et al.* at Johns Hopkins University showed that one can induce stable engraftment in mice after incompatible marrow cell transplants once the mice receive posttransplantation cyclophosphamide (PTCy) without seeing life-threatening GVHD and inducing stable chimerism [6].

### Haploidentical transplantation at Johns Hopkins University, the origins

Based on the aforementioned study by Leo Luznik, O’Donnell *et al.* from Johns Hopkins University, investigated the use of PTCy as part of nonmyeloablative conditioning in partially Haplo-BMT [7]. This is the first report of successful haploidentical transplants with PTCy with acceptable results in humans. Thirteen patients with high-risk hematologic malignancies received conditioning with fludarabine, 30 mg/m2 per day from days −6 to −2, and low-dose total body irradiation (TBI), 2 Gy on day −1. All patients received Cy, 50 mg/kg on day 3, mycophenolate mofetil from day 4 to day 35, and tacrolimus from day 4 to at least day 50. ‘The first cohort received no additional conditioning while the second cohort received Cy with the fludarabine and TBI. Two of the three patients in the first cohort experienced a graft rejection. Eight of the ten patients in the second cohort achieved sustained donor cell engraftment. Two patients with myelodysplastic syndrome rejected the grafts. However, they experienced autologous reconstitution. Six patients were found to develop histologic proven acute GVHD after the transplant, which was unfortunately fatal in one patient. Out of cohort two, six of the ten patients survived, with five in complete remission, including two with graft rejection. This supported the notion that partially HLA-mismatched bone marrow can graft after using nonmyeloablative conditioning. With this study, the field of Haplo-BMT with PTCy was born.

In a follow-up trial, Luznik *et al.* conducted a more extensive study with 68 patients [8]. They found that the incidence rates of grade II-IV acute GVHD (aGVHD) and grade III-IV aGVHD, respectively, were 34% and 6%. Additionally, they found that two doses of Cy resulted in a significantly lower risk of chronic GVHD (cGVHD) *versus* the single dose. Non-relapse mortality (NRM) at one year was found to be 15%, with an incidence of relapse of 51%. Overall survival (OS) and event-free survival (EFS) two years post-transplant were 36% and 26%, respectively. Patients with lymphoid malignancies had improved EFS compared to those with myelogenous malignancies. Their findings supported the use of nonmyeloablative HLA-haploidentical BMT with PTCy, resulting in acceptable rates of fatal graft failure and severe aGVHD or cGVHD. Only 9% experience graft failure, resulting in one fatality. One of the main advantages of this platform is that it was designed from its origins, to allow for ambulatory transplantation, as Luznik *et al.* clearly stated it in the report in 2008 [8].

### Johns Hopkins exports PTCy: multi center studies

The studies above focused on bone marrow grafts; however, peripheral blood stem cell grafts are more widely used. For instances where someone suffers from hematologic malignancies and lacks a related donor match, RIC of unrelated double umbilical cord blood (dUCB) or Haplo-BMT is an effective treatment. Both approaches were tested in 2 simultaneous phase II studies by the Blood and Marrow Transplant Clinical Trials Network [9]. Brunstein *et al.* found that after dUCB treatments, the one-year OS and PFS rates were 54% and 46%, respectively. Comparatively, after the HLA-haploidentical transplant, the one-year OS and PFS rates were 62% and 48%, respectively. While the Haplo-marrow had lower incidence rates of grade II-IV aGVHD at 32%, dUCB was slightly higher at 40%. Rates of cGVHD for dUCB and Haplo-BMT were 25% and 11%, respectively. These findings confirmed that dUCB and Haplo-BMT can be an option when a related donor is unavailable.

In another study conducted by Fuchs *et al.* with the Blood and Marrow Transplant Clinical Trials Network, patients with chemo-sensitive lymphoma or acute leukemia in remission underwent UCB or haploidentical BMT in a random fashion [10]. The conditioning regimen administered was Cy and fludarabine with GVHD prophylaxis of cyclosporine and mycophenolate mofetil (MMF) or PTCy, tacrolimus, and MMF for patients of UCB and Haplo-BMT respectively. UCB had PFS, NRM, and OS of 35%, 18%, and 46% *versus* Haplo-BMT, 41%, 11%, and 57%, respectively. The day 180 incidence rates of cGVHD for UCB and Haplo-BMT patients are 35% and 28%, respectively, while two-year incidence rates were 22% and 26%. However, the 2-year survival favored Haplo-BMT (46 vs 57% *p* = 0.04) [10].

### Haplo-BMT for non-malignant indications

While BMT is commonly associated with treatment for cancerous conditions, it has also been proven to successfully treat non-cancerous conditions such as sickle cell disease, thalassemia, and aplastic anemia. While allogenic BMT is a curative treatment for sickle cell disease, this is not widely used due to the lack of HLA-matched donors. Therefore, in the case of no available HLA-matched donor, a RIC BMT with a haploidentical donor could be an option. Bolaños-Meade *et al.* from Johns Hopkins University reported on a phase I-II study using Haplo-BMT after RIC using PTCy [11]. In this study, 17 patients were transplanted (14 receiving HLA-haploidentical and 3 receiving HLA-matched related donors), and 11 of these patients were engrafted. At the time of the report, ten patients were asymptomatic, and six were off immunosuppressants. These results suggested that RIC conditioning with PTCy expands the donor pool, allowing greater accessibility for patients wishing a BMT. However, 43% of the haploidentical transplants resulted in graft failure.

In a follow-up study by the same group at Johns Hopkins University, aiming to solve the problem of high graft failure rates, a new hypothesis was proposed stating that higher doses of total body irradiation (TBI) would lower these rates [12]. With a GVHD regimen of PTCy, sirolimus, and MMF, 12 sickle cell and five beta-thalassemia patients received anti-thymocyte globulin, fludarabine, Cy, and 4 Gy TBI (instead of 2 Gy). Among all the patients, only one experienced graft failure. Five experienced aGVHD and three cGVHD, but all with complete recovery. Fourteen patients could discontinue immunosuppression, with one still dependent on transfusions. In finding that increasing TBI almost eliminated graft failure, they solved the issue of graft failure.

Another study by DeZern *et al.* at Johns Hopkins University aimed to treat severe aplastic anemia (SAA) with haploidentical BMT with PTCy [13]. Typically, SAA treatment involves BMT from an HLA identical donor to restore hematopoiesis. HLA-haploidentical donors were used after RIC in combination with PTCy to offer treatment to patients without matched donors. They treated three groups, including 27 with SAA, 20 relapse/refractory (R/R), and 17 treatment naïve (TN). The median time for neutrophil recovery was 17 days. Overall, graft failure (GF) was 11%, with one patient in the R/R group and 3 in the TN group receiving 2 Gy TBI. None of the patients receiving 4 Gy had GF. Two patients with GF contracted an infection, and two patients received a second, curative Haplo-BMT. The OS at one and two years was 94%. Rates for grades II-IV: aGVHD at day 100 was 11%, and cGVHD at two years was 8%. After using the same treatment as that used for patients with sickle cell, all 20 R/R patients were alive, disease-free, with no evidence of active GVHD. While TN patients had higher rates of GVHD, a TBI dose of 4 Gy was found to support durable engraftment. Nonmyeloablative Haplo-BMT in TN SAA could allow for the removal of barriers to BMT and curative treatment even for newly diagnosed patients [14,15].

### Comparisons between haploidentical BMT and other types of transplants

A study by Ghosh *et al.* on RIC for Lymphomas compared outcomes after Haplo-BMT using PTCy with those of HLA-matched BMT, using CIBMTR data [16]. Patients in the group undergoing Haplo-BMT received either GVHD prophylaxis with PT-Cy with or without a calcineurin inhibitor or mycophenolate mofetil. The group treated with HLA-matched BMT received calcineurin inhibitor-based GVHD prophylaxis. The comparison of critical outcomes between Haplo-BMT and HLA-matched BMT reveals several significant findings. First, neutrophil recovery at 28 days was similar between the two groups, with rates at 95% for Haplo-BMT and 97% for HLA-matched BMT. However, notable differences emerge in 28-day platelet recovery, where Haplo-BMT shows a rate of 63%, compared to a higher rate of 91% for HLA-matched BMT. Regarding GVHD, the incidence of Grade II-IV GVHD at day 100 is similar between Haplo-BMT at 27% and HLA-matched BMT at 25%. In the context of cGVHD at one year, the rates differ significantly, with Haplo-BMT at 12% and HLA-matched BMT at 45%. Examining long-term outcomes, NRM at three years is 15% for Haplo-BMT compared to 13% for HLA-matched BMT. In terms of relapse/progression, Haplo-BMT shows a rate of 37%, and 40% was observed in HLA-matched BMT. Despite these variations, PFS at three years was equivalent between the two groups, standing at 48%. Moreover, OS rates at three years demonstrate no disparity, with Haplo-BMT at 61% and HLA-matched BMT at 62%. They found no significant difference between Haplo-BMT and HLA-matched BMT in terms of NRM, progression/relapse, PFS, and OS. Essentially, Haplo-BMT with PTCy has similar survival outcomes to HLA-matched BMT with a significantly lower risk of cGVHD.

In a study by Kanate *et al.*, researchers evaluated outcomes for patients receiving Haplo-BMT with PTCy, with patients receiving grafts from HLA-matched unrelated donors (URD) with or without antithymocyte globulin (ATG) after receiving RIC [17]. With a median follow-up of 3 years, the 100-day cumulative incidence of GVHD varies among different transplantation approaches: Haplo-BMT showed a rate of 8%, while URD with no ATG has a rate of 12%, and URD with ATG has the highest rate at 17%. The 1-year cumulative incidence of cGVHD on univariate analysis reveals distinct differences: Haploidentical transplantation demonstrates a lower incidence at 13%, whereas URD with no ATG has a substantially higher incidence at 51%, and URD with ATG falls at 33%. The multivariate analysis highlights that the risk of Grade III-IV aGVHD and cGVHD is higher in URD, both with and without ATG, compared to Haplo-BMT. Examining the cumulative incidence of relapse/progression at three years, Haplo-BMT had a rate of 36%, URD with no ATG has a lower rate of 28%, and URD with ATG is 36%. Survival outcomes at three years differ: Haploidentical transplantation has a survival rate of 60%, URD with no ATG 62%, and URD with ATG lower at 50%. Furthermore, NRM and PFS, as analyzed through multivariate analysis, do not show significant differences between haploidentical transplantation and URD with or without ATG. RIC Haplo-BMT with PTCy did not impact negatively early survival outcomes compared with matched URD transplantation and is associated with a significantly reduced risk of cGVHD [10]. This is one of the many studies comparing Haplo-BMT versus other donors, but many of these comparisons have been published showing similar findings.

It has been established that PTCy reduces rates of GVHD. With this in mind, a study by McCurdy *et al.* focused on adults with hematologic malignancies who received T-cell replete bone marrow grafts and Cy after myeloablative HLA-matched related or unrelated, or non-myeloablative Haplo-BMT [18]. They focused on analyzing one-year GVHD-free, relapsefree survival (RFS), and then cGVHD-free, RFS. The first and second rates for myeloablative HLA-matched related were 47% and 53%, respectively. For myeloablative HLA-matched unrelated the rates were 42% and 52%. Finally, non-myeloablative Haplo-BMT saw rates of 45% and 50%. Overall, this indicates that PTCy-based methods of treatment have very similar composite endpoints across a number of factors including, conditioning intensity, donor type, and HLA match.

Oftentimes patients who need a BMT are of advanced age, given that the median age of presentation for many blood cancers is in the seventh decade of life. A study by Kasamon *et al.* at Johns Hopkins University wanted to better understand the effects of age on RIC Haplo-BMT outcomes in patients between 50 and 75 years old [19]. They performed a retrospective analysis on 271 patients suffering from hematologic malignancies. The patients received T-cell replete Haplo-BMT with PTCy. Eighty-four percent of patients presented with intermediate or high/very high-risk disease. The 6-month probability of grade III-IV GVHD and NRM were 3% and 8% respectively. Additionally, they found that the 6-month NRM for those in their 50s, 60s, and 70s were 8%, 9%, and 7% respectively. The corresponding 3-year PFS probabilities for the same age groups were 48%, 45%, and 44%. Finally, the OS was 39%, 35%, and 33% for each group. Furthermore, the 3-year PFS probabilities for acute myeloid leukemia, aggressive non-Hodgkin lymphoma, and indolent or mantle-cell lymphoma were 40%, 39%, and 37%. These results are significant as older patients were found to have a higher risk for grade II-IV aGVHD but not grade III-IV aGVHD or cGVHD. However, there were no significant associations between older age or NRM, relapse, or survival. Overall, they found the NRM of Haplo-BMT with PTCy shows promising results in older patients. In another study, by the same Johns Hopkins University group by Imus *et al.*, on patients aged over 70, who received a Haplo-BMT, RIC conditioning, and PTCy as GVHD prophylaxis they found that the 2-year OS was 53% with 2-year EFS of 43% [20]. Additionally, the day 180 cumulative incidence of NRM was 14% compared to the 2-year cumulative incidence of 27%, and the 2-year cumulative incidence of relapse of 30%. This supports the idea that this course of treatment is safe and successful in older patients.

### Shortening immunosuppression

A prospective trial of RIC Haplo-BMT for patients with hematologic malignancies, by Kasamon *et al.* at Johns Hopkins University, evaluated the safety of early discontinuation of tacrolimus [21]. They hypothesized that discontinuing immunosuppressive drugs earlier than usual could reduce the risk of relapse as well as improve immune reconstitution in patients receiving bone marrow grafts. However, this strategy has the potential to increase the risk of developing GVHD. Patients received T cell-replete bone marrow followed by PTCy, MMF, and tacrolimus. Tacrolimus was specified to stop without a taper at day +90, +60, or +120. The rules concerning safety stopping were contingent on ≥5% GF, ≥10% NRM, or a ≥20% combined incidence of severe acute and chronic GVHD from the tacrolimus stop date through day +180. Of the 47 patients in the +90 arm, 49% stopped tacrolimus on the indicated stop date. Of the 55 patients in the +60 arm, 69% stopped the treatment as defined. Finally, those in both the +60 and +90 arms at day +180, the probability of grade II-IV aGVHD, grade III-IV aGVHD, cGVHD, and NRM were <40%, <8%, <15%, and <10%, respectively. The 1-year GVHD-free RFS was highest in the 60-day arm. These results indicate that stopping tacrolimus at +60 is possible and has acceptable rates of GVHD after RIC Haplo-BMT with PTCy, as well as lower rates of relapse.

A similar study by DeZern *et al.* from Johns Hopkins University, was done but using peripheral blood (PB) stem cell grafts instead of bone marrow [22]. They chose to stop immunosuppression at either day +60 or +90 following RIC PB stem cell transplantation. The primary goal of this study was to understand the safety and possibility of shorter-duration immunosuppression. The patients of this study suffered from either myelodysplastic syndrome, acute myelogenous leukemia (with minimal residual disease or arising from an antecedent disorder), myeloproliferative neoplasms, myeloma, or chronic lymphoblastic leukemia. They found that the shorted immunosuppression was possible in 64% of patients. The remaining 36% were ineligible due to GVHD, early relapse, NRM, patient/ physician preference, or GF. In the day +90 and +60 cessation groups, 58% and 70%, respectively, stopped immunosuppression as planned. After stopping immunosuppression, the median time in which day +90 and +60 patients developed grade II-IV aGVHD was 21 and 32 days respectively. Around one-third of the patients diagnosed with grade II-IV aGVHD resumed the immunosuppression. The cumulative rates of grade III-IV aGVHD were 2% and 7% in the day +90 and +60 cohorts. The incidence of cGVHD at two years was 9% in the day 90 cohort and 5% in the day 60 cohort. Two-year OS was 67% for both day 90 and day 60 groups. The 2-year PFS was 47% for the day +90 cohort and 52% for the day +60 cohort. The GVHD-free, RFS was <35% for both the day +60 and the day +90 cohorts. This also supports the use of reduced duration immunosuppression as it has acceptable safety rates for patients receiving peripheral blood grafts.

### What to do during relapse

People who relapsed when receiving haploidentical transplants with PTCy can get haploidentical donor lymphocyte infusions (DLI) or second transplants with acceptable results. Here, Zeidan *et al.* from Johns Hopkins University described the outcomes of DLI on patients who received Haplo-BMT with PTCy [23]. Thirty percent of patients had a complete response (CR) with a median duration of 11.8 months. At the last visit, eight patients were alive in CR with 6 for over a year out of the original forty. Haploidentical DLI for relapse after Haplo-BMT has rates of toxicity that are deemed acceptable and result in successful responses, similar to those of DLI in matched pairs.

The ability to use mismatched donors increases the donor pool for a second BMT. Imus *et al.* from Johns Hopkins University reported on a study of patients receiving a second BMT after relapse of disease using PTCy as GVHD prophylaxis [24]. Patients were divided into two groups: those who received a second allograft with the same HLA matching, and those who received an allograft with a new mismatched haplotype. In patients whose first allograft was haploidentical, loss of heterozygosity analysis was performed for patients with AML. Then, 40 patients received a second BMT in cases of relapsed hematologic malignancy. For the entire cohort, the median OS was 928 days with median EFS at 500 days. The 4-year OS and EFS were 40% and 36%, respectively. Over the course of two years, the cumulative incidence of NRM reached 27%. At the 100-day mark, the cumulative incidence of grade III-IV aGVHD was 15%. Looking at a two-year span, the cumulative incidence of extensive cGVHD was found to be 22%. The median survival was 552 days (95% CI 376–2950+) in the group transplanted with a second allograft using the same HLA, while it was not reached in the group whose allograft contained a new mismatched haplotype. Overall, a second BMT is effective in increasing survival for those who have relapsed.

In conclusion, Haploidentical transplants are doable, effective, well tolerated, and solve the problem of donor availability for a large number of patients. They had a fascinating historical development, from the bench, by Luznik *et al.*, all the way to registry and randomized studies [6,9,10]. They showed effectiveness in non-cancerous conditions like hemoglobinopathies and aplastic anemia [11–14]. The rates of GVHD are quite similar to those seen in HLA-matched donor transplants that received PTCy, despite the HLA mismatch and support the use of PTCy as a powerful prophylactic agent against GVHD [25]. Of great relevance is the fact that they are useful for patients receiving transplants for special indications such as severe hemoglobinopathies, aplastic anemia, and second transplants. For the immediate future, one of the topics of most interest is the use of lower doses of cyclophosphamide in an attempt to decrease toxicity [26, 27]. While it is of interest to see what prospective trials in this regard will show, one has to remember that in the original study by Luznik *et al.* a higher rate of chronic GvHD was seen in patients receiving 50 mg/kg once compared to those who received two doses [8]. The development of PTCy from the laboratory to the standard of care is a classic story of science translation from the bench to the bedside.
